# Prevalence and trends of transfusion transmitted infections among blood donors in a tertiary care hospital of Assam

**DOI:** 10.1038/s41598-025-97381-w

**Published:** 2025-08-16

**Authors:** Madhumita Das, Samim S. Hoque, Argha Baruah

**Affiliations:** 1 Guwahati Neurological Research Centre Institute of Medical Science, North Guwahati, Assam 781031 India; 2 Blood Centre, All India Institute of Medical Science, Guwahati, 781101 India

**Keywords:** Sero-prevalence, TTI, Blood group, Demography, Antigen, Transfusion, Health care, Medical research

## Abstract

The overall prevalence of transfusion-transmissible infections (TTIs) in India is 1.58%, while in Assam, it stands at 1.2%. The relatively higher prevalence of TTIs in certain geographical regions can be attributed to illiteracy, lack of awareness, inadequate diagnosis due to poor infrastructure, and insufficient research efforts. Additionally, ABO antigens are also considered to be associated with the risk of developing TTIs. Therefore, the present study was designed to investigate the trend and sero-prevalence of TTIs among blood donors and to evaluate the possible association between TTIs and ABO blood groups. This retrospective observational study was conducted at a tertiary care hospital in Assam over a span of eight and a half years, from June 2015 to December 2023. The medical records of blood donors were reviewed without consideration of age, ethnicity, or gender. Data on demographic details, blood group, and TTI screening test results were collected and analyzed for the study. The overall prevalence of TTIs in our study was found to be 3.1%, with HCV having the highest prevalence (1.14%), followed by syphilis (1.0%), HBV (0.54%), HIV (0.41%), and malaria (0.01%). An overall upward trend in TTI prevalence was observed, with HCV showing a particularly notable increase. A significant positive association (*p* < 0.05) was identified between donors lacking ABO antigens and TTI positivity, while a negative association was observed in those expressing both A and B antigens. Additionally, the present study found that donors with Rh-negative blood groups had a lower likelihood of TTIs compared to Rh-positive donors. The relatively higher prevalence of TTIs highlights a lack of public awareness, insufficient knowledge, and inadequate counselling within the studied population.

**Supplementary Information:**

The online version contains supplementary material available at 10.1038/s41598-025-97381-w.

## Introduction

Blood serves as an ideal medium for transmitting communicable diseases, making blood transfusion safety a critical concern in developing countries like India, where the prevalence of infectious agents among blood donors remains high^1,2^. Transfusion-transmissible infections (TTIs) are infections that can be passed from donor to recipient through the parenteral administration of blood or blood products, posing a significant threat to transfusion recipients^3^. Among the diseases classified as TTIs, the most prevalent are hepatitis B virus (HBV), hepatitis C virus (HCV), human immunodeficiency virus (HIV-I/II), syphilis, and malaria, all of which are required to be screened before blood transfusion^4–6^. TTIs are associated with severe conditions such as acquired immunodeficiency syndrome (AIDS), hepatitis, cirrhosis, and hepatocellular carcinoma, which can lead to long-term health complications^7–9^. Beyond the medical impact, TTIs also have lasting consequences for families and communities, as infected individuals may unknowingly transmit the disease during its asymptomatic phase^2,10^.

Every year, nearly 30 million units of blood and blood components are transfused in India. Therefore, if blood transfusion safety guidelines are not strictly followed, there is a significant risk of developing TTIs from this blood components^7–9^. In India, the overall prevalence rates of HBV, HCV, HIV, syphilis, and malaria are reported to be 0.87%, 0.34%, 0.14%, 0.17%, and 0.06%, respectively. Comparatively, in Assam, the reported prevalence rates are 0.54% (HBV), 0.24% (HCV), 0.12% (HIV), 0.3% (syphilis), and 0.03% (malaria). The total prevalence of TTIs in India stands at 1.58%, while in Assam, it is slightly lower at 1.2%.^6^

It has been observed that there is a relatively higher endemicity of TTIs in tribal areas, which may be attributed to illiteracy, lack of awareness, improper diagnosis due to poor healthcare resources, and inadequate research across various geographical regions^9^. Blood group antigens represent the polymorphic characteristics of individuals^6^, and different blood groups have been reported to be associated with varying susceptibility to pathogenic organisms^4–6^. Research also suggests that an individual’s susceptibility to certain pathogens may be influenced by variations in blood group antigen expression^6,11^. Microorganisms or ecological entities resembling A/B-antigens may trigger the formation of ABO antibodies, which target microbes with ABO-active antigens. Depending on blood group type, the body’s innate immune response to infection may vary^6,12–14^. Blood group antigens are also reported to act as pseudo-receptors, potentially serving as ligands for certain microorganisms. For instance, Plasmodium species bind to the Duffy blood group antigen^6,15^. It has also been reported that microorganisms causing TTIs cannot attach to polysaccharides if ABO antigens are present, whereas cells lacking these antigens are at a higher risk of acquiring TTIs^6,12,16^.

Therefore, the present study was designed to.

1. Analyze the demographic variables among the blood donor population.
2. Explore the trend and sero-prevalence of TTIs among voluntary and replacement blood donors.
3. Investigate any association of TTIs with blood groups through a comprehensive retrospective analysis conducted over eight and a half years (June 2015 to December 2023) at a tertiary care hospital in Assam.

The findings aim to assist in evaluating and improving healthcare services, ensuring safer blood transfusions, and providing essential data for policymaking regarding blood safety. Additionally, this study seeks to provide baseline information for further scientific research and to assess the risk versus benefit of blood transfusion.

## Materials and methods

The present retrospective study was conducted at the Blood Centre of Guwahati Neurological Research Centre Institute of Medical Science (GNRC IMS), North Guwahati, Assam over a span of eight and a half years, from June 2015 to December 2023. It was an observational record analysis study comprising a sample size of 35,000 medical records of blood donors, which were scrutinized, irrespective of age, ethnicity, or gender. Only data related to blood grouping, TTI screening test results, and the demographic details of blood donors, as recorded in the GNRC IMS Blood Centre inventory registers, were used for this study.

The study was conducted with the approval of the institutional ethics committee and all methods complied with the relevant guidelines and regulations of the National Blood Transfusion Council. As it was a retrospective study design and no additional blood samples were obtained from donors, the ethics committee waived the requirement for informed consent. However, before blood donation, informed consent had been obtained from all participating blood donors.

The study population consisted of apparently healthy individuals who visited the blood centre with the intention of donating blood over the past nine years, either voluntarily or as replacement donors. Voluntary donors are motivated individuals who donate blood or blood components at regular intervals of their own volition, without receiving any remuneration. In contrast, replacement donors are family members, friends, or relatives of the patients in need.

All blood donors (voluntary and replacement) underwent routine counselling and screening before donating blood, as per the standard operating procedure (SOP). Only those donors who were deemed healthy and met the inclusion criteria were allowed to donate blood.

### Inclusion criteria

The donor inclusion criteria were as follows:

- Age: For first time donors: 18 to 60 y and repeat donors: 18 to 65 y.
- Weight: > 45 kg.
- Haemoglobin level: 12.5 to 16.5 g/dl.
- Pulse - between 50 and 100/minute with no irregularities.
- Blood Pressure -Systolic 100–180 mm Hg and Diastolic 50–100 mm Hg.
- No history of HBV, HIV, HCV, sexually transmitted diseases, surgery, asthma, pregnancy, lactation and any pathological conditions.
- No recent history of malaria and typhoid fever.
- No history of high- risk behaviours for HIV, hepatitis B/C, and syphilis.
- Past three months - not donated blood or been treated for malaria.
- No history of vaccinations (non-live for past 14 days and live attenuated for 28 day).
- Past 2 weeks - taken any antibiotics or any other medications.
- Past 24 h - taken alcoholic beverages.

### Exclusion criteria

Professional blood donors and individuals who failed to fulfil the blood donation criteria were excluded from this study.

### Serological testing

Donors’ blood samples were collected and tested following all biosafety precautions and infection control protocols. ABO and Rh blood grouping was performed using the column agglutination test with the Gel card method (ABO/RhD Forward/Reverse Grouping Confirmation Card, Matrix, Tulip Diagnostics Pvt. Ltd, Goa, India) following the manufacturer’s instructions. The detection of ABO blood groups is based on the existence of A and B antigens on the surface of red blood cells (RBCs) and the presence of corresponding anti-A or anti-B antibodies in the serum. All donors’ serum samples were screened for major TTIs including HIV, HBV, and HCV using the enhanced chemiluminescent immunoassay technique on VITROS immunodiagnostic system (Ortho-Clinical Diagnostics, USA). Additionally, a rapid chromatographic immunoassay was performed for the detection of syphilis and malaria using test kits from ASPEN, Hangzhou Biotest Biotech Co. Ltd., China and J. Mitra & Co. Pvt. Ltd., New Delhi, India. Testing was conducted for all donors after donation but before blood transfusion. All reactive samples were re-tested in duplicate to confirm the results before releasing them.

### Statistical analysis

For standard statistical analysis, Microsoft Office Excel and SPSS version 21.0 software were used. The sero-prevalence of HBV, HCV, HIV, MP, and syphilis was expressed as percentages for the entire study group, categorized by blood group and various demographic variables. Descriptive statistics, including frequency, percentage, mean, and standard deviation, were used to summarize donor characteristics.

Chi-square or Fisher’s exact test was used for categorical variables, and the Student’s t-test was applied for the comparison of means, depending on the normality assumption. The proportion test was used to assess differences in the prevalence of proportions. A p-value less than 0.05 was considered statistically significant at a 95% confidence interval. Statistical significance was denoted as follows: ‘*’ (*p* < 0.05), ‘**’ (*p* < 0.01), ‘***’ (*p* < 0.001), and ‘****’ (*p* < 0.0001).

### Ethical considerations

Present study was approved by the institutional ethics committee of Institute of Neurological Sciences, GNRC Complex, Dispur, Guwahati (Vide Reg No-ECR/778/Inst/AS/2015/RR-22, Drugs Controller General of India; Reg No-EC/NEW/INST/2023/3358, Department of Health & Family Welfare) by the approval number- Ref No: EC/INS/2024-25/006, dated 30/04/24.

## Results

### Demographic details of the blood donors

In the present study, data from a total of 31,931 blood donors including 31,598 (98.96%) males and 333 (1.04%) females from June 2015 to December 2023 were screened. Of these, 31,338 (98.14%) were replacement donors, while the remaining 593 (1.86%) were voluntary donors. The percentage of female donors was significantly lower across all studied groups. The mean age, weight, and hemoglobin (Hb) level of the blood donors were 31.97 ± 6.37 years, 69.56 ± 6.88 kg, and 13.6 ± 0.85 g%, respectively, with no significant differences observed between replacement and voluntary donors. The majority of the blood donors (92.47%) belonged to the 18–40 years age group, with the highest proportion (31.71%) in the 26–30 years age group, compared to only 7.54% in the 41–55 years age group. Among replacement donors, 64% were from rural areas, whereas 68.13% of voluntary donors were from urban areas. In terms of religion, almost 75% of donors were from the Hindu community, followed by Islamic (21.13%), Christian (1.51%), Sikh (1.32%), and Buddhist (0.99%) communities. The most common blood group among donors was O-Positive (37.03%), followed by B-Positive (30.23%), A-Positive (22.85%), and AB-Positive (7.05%). Only 2.84% of donors were Rhesus-negative (Table 1).

### Demographic details of TTI-reactive donors

Out of a total of 31,931 blood donors, 949 (2.97%) tested positive for at least one transfusion-transmissible pathogen. The positivity rate was relatively higher among males than females (2.97% vs. 0.32%), in the 18–40 years age group compared to other age groups (2.52% vs. 0.44%), among rural compared to urban inhabitants (1.84% vs. 1.14%), among Hindus compared to other religions (2.16% vs. 0.8%), and among Rhesus-positive donors compared to Rhesus-negative donors (2.88% vs. 0.09%). However, these differences in occurrence were not statistically significant. The blood group with the highest positivity rate was O-Positive (1.08%), followed by B-Positive (0.88%), A-Positive (0.68%), and AB-Positive (0.24%). There were no significant differences in the mean age, weight, or Hb levels between replacement and voluntary blood donors (Table 2).

### Prevalence of TTIs

The overall prevalence of TTIs in the study was observed to be 3.1%. Among the TTIs, HCV (1.14%) had the highest prevalence, followed by syphilis (1.0%), HBV (0.54%), HIV (0.41%), and malaria (0.01%). A total of 39 donors (0.12%) tested positive for multiple (mixed) TTIs. The multiple TTI positivity rate was higher among Rhesus-negative donors (0.22%) compared to Rhesus-positive donors (0.12%), although the difference was statistically insignificant (*p* = 0.86). The highest prevalence of TTIs was observed in the year 2021 (4.18%), followed by 2023 (4.15%), 2018 (3.17%), 2022 (2.98%), 2020 (2.68%), 2019 (2.37%), 2016 (2.29%), 2017 (2.12%), and 2015 (1.54%). In terms of individual TTIs, the highest prevalence of HBV (0.66%) and HCV (1.68%) was observed in 2023, HIV (0.69%) in 2018, and syphilis (1.58%) in 2021 (Table 3).

### Prevalence of TTIs among studied blood groups

Out of the total 989 TTI-positive cases (including the mixed infections), A, AB, B, O, Rhesus-positive, and Rhesus-negative blood donors were 233 (3.08%), 84 (3.57%), 298 (3.02%), 374 (3.08%), 958 (3.09%), and 31 (3.41%), respectively. The overall TTI reactivity was observed to be higher among Rhesus-negative blood donors compared to Rhesus-positive donors (3.41% vs. 3.09%), particularly in cases of syphilis infection (1.87% vs. 0.97%). Of the 173 (0.54%) HBV-positive donors, the percentages of A, AB, B, O, Rhesus-positive, and Rhesus-negative donors were 38 (0.5%), 10 (0.43%), 49 (0.5%), 76 (0.63%), 169 (0.54%), and 4 (0.44%), respectively. A total of 364 (1.14%) blood donors were tested positive for HCV, with 84 (1.11%) A, 27 (1.15%) AB, 103 (1.04%) B, 150 (1.24%) O, 356 (1.15%) Rhesus-positive, and 8 (0.88%) Rhesus-negative donors. The percentages of A, AB, B, O, Rhesus-positive, and Rhesus-negative blood group among HIV-positive donors were 33 (0.44%), 15 (0.64%), 42 (0.43%), 41 (0.34%), 129 (0.42%), and 2 (0.22%), respectively. Only 2 (0.02%) blood donors of the O Rhesus-positive blood group were tested positive for malaria. Of the total 319 (1%) syphilis-positive donors, the proportions of A, AB, B, O, Rhesus-positive, and Rhesus-negative donors were 78 (1.03%), 32 (1.36%), 104 (1.05%), 105 (0.86%), 302 (0.97%), and 17 (1.87%), respectively. The TTI positivity rate was higher among donors with the AB blood group (3.57%) compared to others. Regarding the prevalence of individual TTIs, syphilis showed the highest prevalence rate of 1.36%, primarily involving AB group blood donors, followed by HCV infection (1.24%), which mostly affected O group donors. Donors with the O blood group demonstrated a somewhat higher prevalence (0.16%) of multiple infections compared to other blood groups (Table 3).

### Trends of TTIs

The results of the present study revealed a consistent increase in the cumulative frequency of overall TTI positivity from 2015 to 2023, with the exception of 2022, when a slight decline in frequency was observed. Notably, the prevalence of TTIs showed a general upward trend, with significant increases in 2016, 2018, and 2021.

**Fig. 1:**
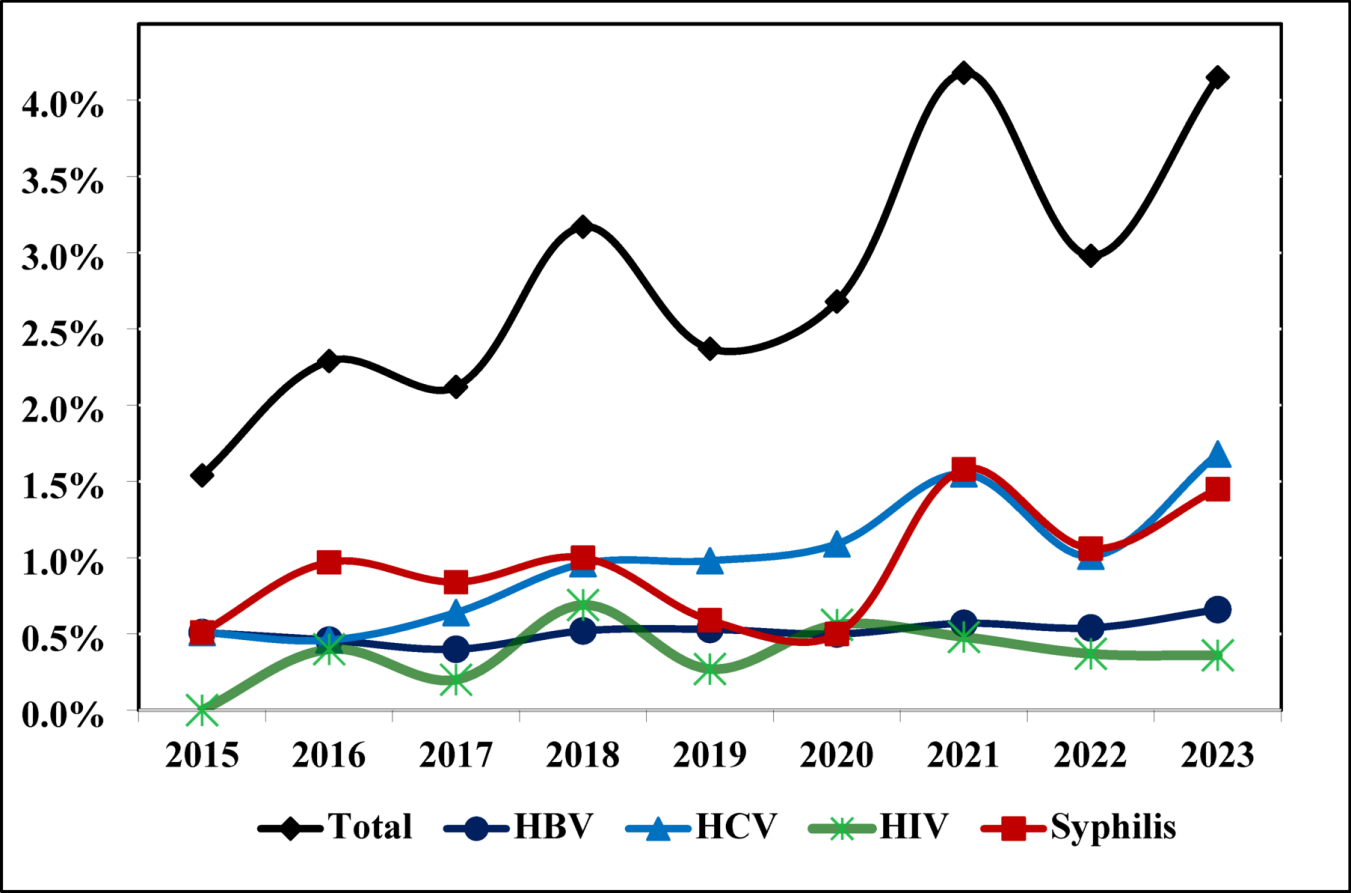
Graphical representation of the trend of TTI prevalence.

In particular, HCV prevalence exhibited a clear rise, increasing from 0.51% in 2015 to 1.68% in 2023, despite slight decreases in 2016 (0.46%) and 2022 (1.01%). On the other hand, HBV prevalence remained relatively stable throughout the study period, while other TTIs did not follow a discernible trend (Fig. 1).

A total of 173 (0.54%) blood donors tested positive for HBV. Among them, the distribution by blood group was as follows: A (38, 0.5%), AB (10, 0.43%), B (49, 0.5%), O (76, 0.63%), Rhesus-positive (169, 0.54%), and Rhesus-negative (4, 0.44%). For HCV, 364 (1.14%) blood donors were positive. Their distribution was: A (84, 1.11%), AB (27, 1.15%), B (103, 1.04%), O (150, 1.24%), Rhesus-positive (356, 1.15%), and Rhesus-negative (8, 0.88%). A total of 131 (0.41%) blood donors were positive for HIV-I/II, distributed as follows: A (33, 0.44%), AB (15, 0.64%), B (42, 0.43%), O (41, 0.34%), Rhesus-positive (129, 0.42%), and Rhesus-negative (2, 0.22%). Only 2 blood donors with the O Rhesus-positive blood group tested positive for malaria. A total of 319 (1.0%) blood donors were positive for syphilis. Their distribution was: A (78, 1.03%), AB (32, 1.36%), B (104, 1.05%), O (105, 0.86%), Rhesus-positive (302, 0.97%), and Rhesus-negative (17, 1.87%) (Table 3).

### Association of individual TTI with different blood groups

The relative percentages of HBV (43.93% vs. 5.78%), HCV (41.21% vs. 7.42%), HIV (31.3% vs. 11.45%), and syphilis (32.92% vs. 10.03%) positivity were significantly higher in donors without any ABO antigens (O group) compared to donors with both A and B antigens (AB group). A statistically significant association (*p* < 0.05) was observed between donors lacking ABO antigens and those possessing both A and B antigens across all TTI-positive cases. The highest positivity rates for TTIs were observed in donors without any antigens, except for HIV, where donors with the B antigen exhibited the highest positivity rate (32.06%), slightly surpassing those without any antigens (31.3%). However, this difference was minimal. In Rhesus negative blood donors, percentage of HBV, HCV and HIV positivity was slightly lower (2.31%, 2.2%, 1.53%, respectively) than the HBV, HCV and HIV negative donors (2.85% in all). Whereas, in Rhesus positive donors, percentage of HBV, HCV and HIV positivity was higher (97.69%, 97.8%, 98.47%, respectively) than the HBV, HCV and HIV negative donors (97.15% in all), but the association was not statistically significant. For syphilis, a divergent pattern was observed between Rhesus-negative and Rhesus-positive blood donors among both syphilis positive and negative cases, but this association was also not statistically significant (*p* > 0.05). The magnitude of TTIs in Rhesus-negative donors (2.84%) was lower compared to Rhesus-positive donors (97.16%) (Table S1, Supplementary File).

### Multiple (Mixed) TTI positivity

Among the 39 donors who tested positive for multiple TTIs, all were replacement donors (Table S2, Supplementary File).

- *HBV and HCV dual positivity*: 12 donors (0.038%) were positive, 10 (0.031%) of whom belonged to the O Rhesus-positive blood group.
- *HBV and syphilis dual positivity*: 2 donors (0.006%) were positive: one with A Rhesus-positive and one with O Rhesus-positive blood groups.
- *HCV and HIV dual positivity*: 6 donors (0.019%) were positive: 1 with A Rh-positive, 3 with B Rh-positive, and 2 with O Rh-positive blood groups.
- *HCV and syphilis dual positivity*: 2 donors (0.006%) were positive: one with AB-positive and one with B Rhesus-negative blood groups.
- *HIV and syphilis dual positivity*: 16 donors (0.05%) were positive: 5 with A-positive, 2 with AB-positive, 3 with B-positive, and 6 with O-positive blood groups.
- Triple TTI positivity (HCV, HIV, and syphilis): Only 1 donor (0.003%) was found to have triple infections, belonging to the B-positive blood group.

### TTI positivity rate by demographic variables

When analyzing the frequency of individual TTIs by various demographic criteria, significant associations were observed with age group (*p* < 0.0001) and locality (*p* = 0.0008). Other demographic variables such as gender, religion and donor type failed to demonstrate any significant associations with the TTI positivity rate. Out of a total of 333 female donors, only one donor, having blood group “O” Rhesus-positive, tested positive for TTI (syphilis).

### Age group

The prevalence of TTIs showed a decreasing trend with increasing age until 40 years, followed by a rise among middle-aged donors, with a sudden peak in the 46–50-year age group (14.63%). This age group exhibited particularly high positivity rates for HBV (2.09%) and syphilis (9.85%) compared to other age groups. Conversely, HCV (3.07%) and HIV (0.8%) were most prevalent in the 18–25-year age group. The 36–40-year age group demonstrated the lowest TTI prevalence (1.54%), which was consistent across all TTIs: HBV (0.39%), HCV (0.36%), HIV (0.14%), and syphilis (0.65%).

### Locality

Except for syphilis, other TTIs were more prevalent among urban blood donors compared to rural donors (3.23% vs. 3.02%).

### Religious communities

Donors from the Islamic community had the highest TTI positivity rate (3.35%), followed by Buddhist (3.16%), Sikh (3.09%), Hindu (3.03%), and Christian donors (2.9%).

- HBV prevalence was highest among Christian donors (1.04%).
- HCV prevalence was highest among Sikh donors (1.43%).
- HIV (0.52%) and syphilis (1.14%) were most prevalent among Islamic donors.

### Donation type

The prevalence of TTIs was higher, though not statistically significant, among replacement donors (3.14%) than voluntary donors (0.84%). All five TTIs were more prevalent in replacement donors compared to voluntary donors (Table 4).

Multivariate analysis (Table S3, Supplementary File) revealed a statistically significant association between the overall prevalence of TTIs and both age (*p* < 0.0001) and locality (*p* = 0.01).

## Discussion

Consistent with some previous studies^6,10,17–21^, the present study also observed a predominance of male blood donors, with nearly 99% (31,598/31,931) of donors being male. This male predominance may be attributed to societal myths, such as the belief that ‘males are stronger and healthier than females and thus more suitable as blood donors.’ Additionally, cultural stigmas suggesting that blood donation might jeopardize women’s health—particularly due to regular blood loss from menstruation—may deter female participation. Physiological conditions such as pregnancy and breastfeeding further contribute to this disparity^10,22–24^. Another significant factor for the lower female donor rate is the higher rejection rate during the screening process, often due to anaemia among females. However, other studies have reported nearly equal proportions of male and female blood donors^17^. In the present study, the majority of donors (92.47%) were aged between 18 and 40 years, suggesting that younger individuals are more educated, aware, and more likely to meet the selection criteria for blood donation compared to older age groups.

In spite of the World Health Organization’s (WHO) recommendation for voluntary blood donation (80–100%) to ensure safe transfusion^10,22,25^, we observed only 1.86% (593/31,931) voluntary donors compared to 98.14% (31,338/31,931) replacement donors in our study, indicating a very low rate of voluntary donations in this region, contrary to the findings of some other studies^10,21,26–28^. The apparent reasons for this scenario may include fear and apprehension associated with blood donation and a lack of basic health education and awareness among the general population. A comparatively higher proportion of voluntary donors from urban areas (68.13%) compared to rural areas (31.87%) highlights the significant lack of awareness in rural populations compared to their urban counterparts. Conversely, in the case of replacement donors, 64% hailed from rural areas compared to 36% from urban areas. This disparity can be attributed primarily to the patient demographics in our hospital, as the majority of admitted patients were from rural areas, resulting in a higher proportion of rural replacement donors.

According to a published report on the assessment of blood banks in India (2016), the overall prevalence of TTIs in India was 1.58%, with the prevalence of HBV, HCV, HIV, malaria, and syphilis being 0.87%, 0.34%, 0.14%, 0.06%, and 0.17%, respectively^29^. However, the present study observed a much higher overall prevalence of TTIs (3.1%), attributed to a significantly higher prevalence of HCV (1.14%), HIV (0.41%), and syphilis (1.0%) compared to the report. Conversely, a decreased prevalence of HBV (0.54%) and malaria (0.01%) was observed in our study. In contrast to the prevalence of TTIs reported in other parts of India, such as Ahmedabad (0.58%),^30^ Central India (1.43%),^31^ Gujarat (0.77%),^32^ Odisha (1.89%),^33^ Ranchi (1.59%),^34^ and Telangana (0.96%),^35^ our study observed a comparatively higher prevalence (3.1%). The highest prevalence of TTIs among the Indian states was observed in Puducherry (3.13%),^29^ which was comparable to our results. In contrast, a much higher prevalence of TTIs has been reported from some other countries, e.g., Brazil (4.04%),^36^ Burkina Faso (24%),^17^ Eastern Ethiopia (7.06%),^37^ Northwestern Ethiopia (5.43%),^38^ Equatorial New Guinea (18.7%),^17^ Mozambique (37.39%),^17^ Peshawar, Pakistan (5.33%),^39^ and the western region of Saudi Arabia (7.93%).^17^

In the present study, the highest prevalence among all the TTIs was observed for HCV (1.14%), followed by syphilis (1.0%), whereas most of the published data revealed a higher prevalence of HBV among blood donors^6,10,17,30–38^. Similar to our results, higher prevalence rates of HCV (among all the TTIs) were reported in Chandigarh, Punjab, Manipur, and Mizoram, while Arunachal Pradesh showed a higher prevalence of syphilis^29^.

The higher prevalence of TTIs observed in the current study can be attributed to several factors, including a lack of public awareness, lax adherence to guidelines and SOPs, inadequate pre-donation counselling, insufficient medical screening to identify high-risk donors, and the use of highly sensitive screening tests. Despite the higher overall prevalence of TTIs, the lower prevalence of HBV in our study compared to most previous studies may be due to community-based awareness campaigns emphasizing the chronic nature of the disease and its transmission. Additionally, the rigorous hepatitis B vaccination drive conducted by the Government of India among the general population likely contributed to the reduced prevalence of HBV observed in our study^35^.

The detection of multiple (mixed) TTIs in 39 donors (0.12%) could be attributed to several factors, primarily serological cross-reactivity due to structural similarities among certain viral and bacterial antigens, leading to false-positive reactions. For example, HCV and HBV share antigenic regions, which may cause cross-reactivity, while syphilis and HIV may exhibit similar issues due to shared epitopes. Additionally, variations in test kits and methodologies can influence results, as some assays are more prone to cross-reactivity than others. High-sensitivity tests may detect low levels of antibodies, leading to false positives in donors with past exposure but no active infection. Moreover, TTIs such as HIV, HBV, HCV, and syphilis share common transmission routes, including blood transfusion, unprotected sexual contact, and intravenous drug use, increasing the likelihood of true co-infections in certain high-risk populations.

The exception observed in 2022, disrupting the consistent increase in cumulative TTI positivity from 2015 to 2023, may likely be attributed to the impact of the COVID-19 pandemic.

Several previous studies have reported no significant association between TTI infection and ABO or Rhesus blood groups^32,33,37^. However, some studies have indicated a higher risk of developing TTIs in O blood group donors^17,39^, while AB blood group donors were reported to have the lowest risk^17^. Contradictory findings, such as no association between O blood group and TTIs, have also been documented^38^.

In the current study, a significantly positive association was observed between donors lacking ABO antigens (O blood group) and TTIs, whereas a negative association was noted with donors expressing both A and B antigens (AB blood group). Consistent with some other studies^40,41^, the present study also found that donors with Rh-negative blood groups had a lower likelihood of TTIs compared to Rh-positive donors.

This study underscores the need for targeted awareness campaigns, stricter donor screening, and improved adherence to safety protocols to enhance blood donation and transfusion safety. The high TTI prevalence highlights gaps in public health education, while the observed blood group associations warrant further research into infection susceptibility. Strengthening community outreach and screening measures can improve donor participation and ensure a safer blood supply.

## Conclusion

The remarkably higher prevalence of TTIs (3.1%) compared to national averages (1.58%) observed in this study, primarily driven by elevated rates of HCV (1.14%), syphilis (1.0%), and HIV (0.41%), underscores a critical gap in public awareness and insufficient knowledge about TTIs among blood donors. The relatively lower prevalence of HBV (0.54%) in our study, as opposed to previous national reports, may reflect the impact of widespread hepatitis B vaccination and community-level awareness initiatives. The study also reinforces emerging evidence of a significant association between blood group phenotypes and TTI prevalence. Our findings indicate that donors with the O blood group, which lacks A and B antigens, demonstrated a higher risk of TTIs, whereas donors with the AB blood group showed a protective association. These results align with previous studies suggesting a potential immunological basis for differential susceptibility to infections based on blood group antigens. However, further research with larger, multicentric datasets is necessary to validate these associations and explore the underlying biological mechanisms.

### Limitations of the study

The current study has limitations beyond its retrospective nature, which could introduce inaccuracies due to incomplete or inconsistent documentation. Additionally, several other limitations of this study are outlined below. Addressing these issues could provide a more comprehensive understanding of TTI prevalence and trends, thereby enhancing blood safety and public health strategies in the region:

1. The study was conducted in a single tertiary care hospital blood centre, which may not fully represent the broader population of Assam limiting the generalizability of the findings.
2. The inclusion of only eligible blood donors may exclude data on high-risk individuals, potentially underestimating the actual prevalence of TTIs in the general population.
3. The study primarily utilized screening tests for TTIs, which, while sensitive, may not differentiate between false positives and true positives without confirmatory testing like NAT (Nucleic acid testing).
4. The study focused on specific TTIs (e.g., HCV, HBV, HIV, malaria and syphilis), excluding other emerging infections that could pose risks to blood safety.
5. The slight drop in TTI prevalence in 2022 could be influenced by external factors like reduced blood donations during the COVID-19 pandemic. Such disruptions may not accurately reflect true epidemiological trends.
6. A higher rate of replacement donors is another limitation of the present study as it can impact blood safety and availability as well as contradicts global best practices.
7. A key limitation of this study is the uncertainty surrounding the 39 donors who tested positive for mixed infections (TTI), which may have led to an overestimation or misinterpretation of the prevalence rate. It remains unclear whether these cases represent true co-infections or are the result of serological cross-reactivity in the diagnostic assays. Future studies should address these limitations by incorporating more specific diagnostic techniques to minimize cross-reactivity and enhance the accuracy of mixed TTI detection.
8. The stark gender disparity limits the study’s ability to analyze TTI prevalence and risk factors among female donors without which any conclusions regarding gender-specific risk factors remain inconclusive.

**Table 1:** Demographic details of the blood donors of GIMS blood centre from Jun’15 to Dec’23.

Demographic details		Total donors	Replacement donors	Voluntary donors
Donor; n(%)	Overall	31,931(100%)	31,338(98.14%)	593(1.86%)
Gender; n(%)	Female	333(1.04%)	290(0.93%)	43(7.25%)
	Male	31,598(98.96%)	31,048(99.07%)	550(92.75%)
Age gps; n(%)	18-25 y	4882(15.29%)	4776(15.24%)	106(17.88%)
	26-30 y	10,124(31.71%)	9936(31.71%)	188(31.7%)
	31-35 y	6515(20.4%)	6402(20.43%)	113(19.06%)
	36-40 y	8004(25.07%)	7866(25.1%)	138(23.27%)
	41-45 y	1868(5.85%)	1834(5.85%)	34(5.73%)
	46-50 y	335(1.05%)	326(1.04%)	09(1.52%)
	51-55 y	203(0.64%)	198(0.63%)	05(0.84%)
G. Location; n(%)	Rural	20,244(63.4%)	20,055(64%)	189(31.87%)
	Urban	11,687(36.6%)	11,283(36%)	404(68.13%)
Religion; n(%)	Buddhist	316(0.99%)	298(0.95%)	18(3.04%)
	Christian	482(1.51%)	452(1.44%)	30(5.06%)
	Hindu	23,964(75.05%)	23,501(74.99%)	463(78.08%)
	Islamic	6748(21.13%)	6678(21.31%)	70(11.8%)
	Sikh	421(1.32%)	409(1.31%)	12(2.02%)
Blood gp; n(%)	A Negative	268(0.84%)	261(0.83%)	07(1.18%)
	A Positive	7297(22.85%)	7176(22.9%)	121(20.4%)
	AB Negative	102(0.32%)	98(0.31%)	04(0.67%)
	AB Positive	2250(7.05%)	2211(7.06%)	39(6.58%)
	B Negative	218(0.68%)	210(0.67%)	08(1.35%)
	B Positive	9652(30.23%)	9463(30.2%)	189(31.87%)
	O Negative	320(1.0%)	314(1.0%)	06(1.01%)
	O Positive	11,824(37.03%)	11,613(37.06%)	211(35.58%)
Age (Avg ± SD)	Overall	31.97 ± 6.37 (yr)	31.97 ± 6.37(yr)	31.69 ± 6.63 (yr)
	Female	28.43 ± 4.57 (yr)	28.47 ± 4.48 (yr)	28.19 ± 5.21 (yr)
	Male	32.01 ± 6.38 (yr)	32.01 ± 6.37 (yr)	31.97 ± 6.65 (yr)
Weight (Avg ± SD)	Overall	69.56 ± 6.88 (kg)	69.53 ± 6.89 (kg)	71.35 ± 6.17 (kg)
	Female	58.66 ± 3.86 (kg)	58.54 ± 3.63 (kg)	59.51 ± 5.12 (kg)
	Male	69.67 ± 6.82 (kg)	69.63 ± 6.83 (kg)	71.93 ± 5.75 (kg)
Hb (Avg ± SD)	Overall	13.6 ± 0.85 (g%)	13.6 ± 0.84 (g%)	13.7 ± 0.88 (g%)
	Female	12.9 ± 0.35 (g%)	12.8 ± 0.34 (g%)	12.9 ± 0.44 (g%)
	Male	13.6 ± 0.85(g%)	13.6 ± 0.84 (g%)	13.7 ± 0.88 (g%)

**Table 2:** Demographic details of TTI reactive donors at GIMS blood centre from Jun’15 to Dec’23.

Demographic Details		Total donors	Replacement (R) donors (*n*=31338)	Voluntary (V) donors (*n*=593)	*p* value
		(*n*=31931)			
TTI reactive	Overall	949 (2.97%)	944 (3.01%)	05 (0.84%)	R/V: 0.27
Gender	Female	01 (0.32%)	01(0.32%)	0 (0%)	M/F: 0.14
	Male	948 (2.97%)	943 (3.01%)	05 (0.84%)	
Age Groups	18‒25 y	271 (0.85%)	269 (0.86%)	02 (0.34%)	
	26‒30 y	247 (0.77%)	246 (0.78%)	01 (0.17%)	
	31‒35 y	167 (0.52%)	166 (0.53%)	01 (0.17%)	18‒40/41‒55 y: 0.23
	36‒40 y	122 (0.38%)	122 (0.39%)	0 (0%)	
	41‒45 y	81 (0.25%)	80 (0.26%)	01 (0.17%)	
	46‒50 y	48 (0.15%)	48 (0.15%)	0 (0%)	
	51‒55 y	13 (0.04%)	13 (0.04%)	0 (0%)	
Location	Rural	586 (1.84%)	582 (1.86%)	04 (0.67%)	Rural/Urban: 0.69
	Urban	363 (1.14%)	362 (1.16%)	01 (0.17%)	
Religion	Buddhist	10 (0.03%)	10 (0.03%)	0 (0%)	
	Christian	14 (0.04%)	14 (0.04%)	0 (0%)	
	Hindu	691 (2.16%)	687 (2.19%)	04 (0.67%)	Hindu/Others: 0.43
	Islamic	221 (0.69%)	220 (0.7%)	01 (0.17%)	
	Sikh	13 (0.04%)	13 (0.04%)	0 (0%)	
Blood Group	A Negative	09 (0.03%)	09 (0.03%)	0 (0%)	
	A Positive	216 (0.68%)	214 (0.68%)	2 (0.34%)	
	AB Negative	04 (0.01%)	04 (0.01%)	0 (0%)	
	AB Positive	77 (0.24%)	77 (0.25%)	0 (0%)	(Rh+ve/Rh-ve) 0.1
	B Negative	07 (0.02%)	7 (0.02%)	0 (0%)	
	B Positive	282 (0.88%)	282 (0.9%)	0 (0%)	
	O Negative	09 (0.03%)	09 (0.03%)	0 (0%)	
	O Positive	345 (1.08%)	342 (1.09%)	3 (0.51%)	
Age (Avg ± SD)	Overall	31.25 ± 8.2	31.25 ± 8.2	30.2 ± 9.26	R/V: 0.89
	Female	27± 0.0	27± 0.0	27± 0.0	R/V: 1.0
	Male	31.25 ± 8.2	31.25 ± 8.2	30.2 ± 9.26	R/V: 0.89
Wt (Avg ± SD)	Overall	69.37 ± 7.19	69.37 ± 7.19	69.4 ± 8.93	R/V: 0.99
	Female	56± 0.0	56± 0.0	56± 0.0	R/V: 1.0
	Male	69.38 ± 7.18	69.38 ± 7.18	69.4 ± 8.93	R/V: 0.99
Hb (Avg ± SD)	Overall	13.6 ± 0.85	13.6 ± 0.85	12.9 ± 0.27	R/V: 0.89
	Female	12.6± 0.0	12.6± 0.0	12.6± 0.0	R/V: 1.0
	Male	13.6 ± 0.85	13.6 ± 0.85	12.9 ± 0.27	R/V: 0.89

**Table 3:** Blood group and year wise prevalence (n/%) of TTI reactivity at GIMS blood centre.

Year	Overall (*n*)	HBV	HCV	HIV	Malaria	Syphilis	Mixed	Donor
**2015** A	03/2.01	01/0.67	0	0	0	02/1.34	0	149
AB	02/4.0	0	02/4.0	0	0	0	0	50
B	02/1.16	01/0.58	0	0	0	01/0.58	0	172
O	02/0.93	01/0.47	01/0.47	0	0	0	0	215
RhP	09/1.58	03/0.53	03/0.53	0	0	03/0.53	0	570
RhN	0	0	0	0	0	0	0	16
**Overall**	**09/1.54**	**03/0.51**	**03/0.51**	**0**	**0**	**03/0.51**	**0**	**586**
**2016** A	13/2.73	03/0.63	02/0.42	03/0.63	0	05/1.05	0	477
AB	02/1.92	0	0	01/0.96	0	01/0.96	0	104
B	10/1.9	03/0.57	02/0.38	02/0.38	0	03/0.57	0	527
O	15/2.35	02/0.31	04/0.63	01/0.16	0	08/1.26	01/0.16	637
RhP	38/2.23	08/0.47	08/0.47	07/0.41	0	15/0.88	01/0.06	1702
RhN	02/4.65	0	0	0	0	02/4.65	0	43
**Overall**	**40/2.29**	**08/0.46**	**08/0.46**	**07/0.4**	**0**	**17/0.97**	**01/0.06**	**1745**
**2017** A	09/2.01	01/0.22	04/0.89	0	0	04/0.89	0	447
AB	03/2.65	0	01/0.88	0	0	02/1.77	0	113
B	10/1.52	03/0.46	04/0.61	01/0.15	0	02/0.30	0	659
O	21/2.61	04/0.50	04/0.50	03/0.37	01/0.12	09/1.12	01/0.12	805
RhP	40/2.04	07/0.36	12/0.61	04/0.2	01/0.05	16/0.82	01/0.05	1956
RhN	03/4.41	01/1.47	01/1.47	0	0	01/1.47	0	68
**Overall**	**43/2.12**	**08/0.4**	**13/0.64**	**04/0.2**	**01/0.05**	**17/0.84**	**01/0.05**	**2024**
**2018** A	27/3.42	05/0.63	12/1.52	05/0.63	0	05/0.63	0	790
AB	12/5.56	0	02/0.93	03/1.39	0	07/3.24	01/0.46	216
B	25/3.04	04/0.49	05/0.61	07/0.85	0	09/1.09	0	822
O	28/2.60	06/0.56	09/0.83	05/0.46	0	08/0.74	0	1078
RhP	90/3.16	15/0.53	27/0.95	20/0.7	0	28/0.98	01/0.04	2848
RhN	02/3.45	0	01/1.72	0	0	01/1.72	0	58
**Overall**	**92/3.17**	**15/0.52**	**28/0.96**	**20/0.69**	**0**	**29/1.0**	**01/0.03**	**2906**
**2019** A	34/2.98	08/0.70	13/1.14	05/0.44	0	08/0.70	01/0.09	1140
AB	10/3.19	02/0.64	04/1.28	02/0.64	0	02/0.64	0	313
B	45/2.52	08/0.45	20/1.12	04/0.22	0	13/0.73	0	1788
O	32/1.72	09/0.48	13/0.70	03/0.16	0	07/0.38	0	1863
RhP	114/2.33	26/0.53	49/1.0	12/0.24	0	27/0.55	01/0.02	4902
RhN	07/3.47	01/0.5	01/0.5	02/0.99	0	03/1.49	0	202
**Overall**	**121/2.37**	**27/0.53**	**50/0.98**	**14/0.27**	**0**	**30/0.59**	**01/0.02**	**5104**
**2020** A	32/2.60	05/0.41	18/1.46	06/0.49	0	03/0.24	01/0.08	1233
AB	10/2.72	01/0.27	03/0.82	04/1.09	0	02/0.54	01/0.27	368
B	30/2.10	09/0.63	08/0.56	04/0.28	0	09/0.63	0	1426
O	61/3.16	10/0.52	25/1.29	14/0.72	01/0.05	11/0.57	03/0.16	1933
RhP	132/2.75	25/0.52	54/1.12	28/0.58	01/0.02	24/0.5	05/0.1	4807
RhN	01/0.65	0	0	0	0	01/0.65	0	153
**Overall**	**133/2.68**	**25/0.5**	**54/1.09**	**28/0.56**	**01/0.02**	**25/0.5**	**05/0.1**	**4960**
**2021** A	41/4.23	06/0.62	12/1.24	04/0.41	0	19/1.96	02/0.21	969
AB	07/1.86	01/0.27	03/0.80	0	0	03/0.80	0	377
B	56/4.56	04/0.33	19/1.55	09/0.73	0	24/1.95	02/0.16	1228
O	71/4.40	13/0.81	31/1.92	07/0.43	0	20/1.24	05/0.31	1612
RhP	173/4.21	24/0.58	65/1.58	20/0.49	0	64/1.56	09/0.22	4110
RhN	02/2.63	0	0	0	0	02/2.63	0	76
**Overall**	**175/4.18**	**24/0.57**	**65/1.55**	**20/0.48**	**0**	**66/1.58**	**09/0.22**	**4186**
**2022** A	24/2.29	01/0.1	08/0.76	04/0.38	0	11/1.05	01/0.1	1050
AB	16/3.99	02/0.50	01/0.25	04/1.0	0	09/2.24	0	401
B	42/2.86	10/0.68	13/0.89	06/0.41	0	13/0.89	0	1467
O	62/3.24	13/0.68	27/1.41	04/0.21	0	18/0.94	03/0.16	1911
RhP	138/2.94	26/0.55	48/1.02	18/0.38	0	46/0.98	04/0.09	4692
RhN	06/4.38	0	01/0.73	0	0	05/3.65	0	137
**Overall**	**144/2.98**	**26/0.54**	**49/1.01**	**18/0.37**	**0**	**51/1.06**	**04/0.08**	**4829**
**2023** A	50/3.82	08/0.61	15/1.15	06/0.46	0	21/1.6	03/0.23	1310
AB	22/5.37	04/0.98	11/2.68	01/0.24	0	06/1.46	01/0.24	410
B	78/4.38	07/0.39	32/1.8	09/0.51	0	30/1.68	06/0.34	1781
O	82/3.92	18/0.86	36/1.72	04/0.19	0	24/1.15	07/0.33	2090
RhP	224/4.12	35/0.64	90/1.66	20/0.37	0	79/1.45	15/0.28	5436
RhN	08/5.16	02/1.29	04/2.58	0	0	02/1.29	02/1.29	155
**Overall**	**232/4.15**	**37/0.66**	**94/1.68**	**20/0.36**	**0**	**81/1.45**	**17/0.3**	**5591**
**Total** A	233/3.08	38/0.50	84/1.11	33/0.44	0	78/1.03	08/0.11	7565
AB	84/3.57	10/0.43	27/1.15	15/0.64	0	32/1.36	03/0.13	2352
B	298/3.02	49/0.50	103/1.04	42/0.43	0	104/1.05	08/0.08	9870
O	374/3.08	76/0.63	150/1.24	41/0.34	02/0.02	105/0.86	20/0.16	12,144
RhP	958/3.09	169/0.54	356/1.15	129/0.42	02/0.01	302/0.97	37/0.12	31,023
RhN	31/3.41	04/0.44	08/0.88	02/0.22	0	17/1.87	02/0.22	908
**Overall**	**989/3.1**	**173/0.54**	**364/1.14**	**131/0.41**	**02/0.01**	**319/1.0**	**39/0.12**	**31,931**

**Table 4:** Prevalence rate` of TTIs by demographic variables, and types of donors.

Criteria	Total donors	TTIs	HBV	HCV	HIV	Malaria	Syphilis	*p* value
	*n*(%)	*n*(%)	*n*(%)	*n*(%)	*n*(%)	*n*(%)	*n*(%)	
Gender								
Male	31,598(98.96)	988(3.12)	173(0.55)	364(1.15)	131(0.41)	2(0.006)	318(1.0)	0.7157
Female	333(1.04)	01(0.30)	0(0)	0(0)	0(0)	0(0)	01(0.30)	
Age (y)								
18-25	4882(15.29)	287(5.88)	42(0.86)	150(3.07)	39(0.80)	01(0.02)	55(1.13)	
26-30	10,124(31.71)	262(2.59)	56(0.55)	97(0.96)	37(0.37)	0(0)	72(0.71)	
31-35	6515(20.4)	172(2.64)	27(0.41)	58(0.89)	29(0.45)	01(0.02)	57(0.87)	<0.0001
36-40	8004(25.07)	123(1.54)	31(0.39)	29(0.36)	11(0.14)	0(0)	52(0.65)	
41-45	1868(5.85)	82(4.39)	09(0.48)	19(1.02)	14(0.75)	0(0)	40(2.14)	
46-50	335(1.05)	49(14.63)	07(2.09)	08(2.39)	01(0.30)	0(0)	33(9.85)	
51-55	203(0.64)	14(6.9)	01(0.49)	03(1.48)	0(0)	0(0)	10(4.93)	
Locality								0.0008
Rural	20,244(63.4)	612(3.02)	101(0.50)	230(1.14)	62(0.31)	02(0.01)	217(1.07)	
Urban	11,687(36.6)	377(3.23)	72(0.62)	134(1.15)	69(0.59)	0(0)	102(0.87)	
Religion								
Hindu	23,964(75.05)	726(3.03)	141(0.59)	260(1.08)	91(0.38)	1(0.004)	233(0.97)	
Islamic	6748(21.13)	226(3.35)	22(0.33)	91(1.35)	35(0.52)	1(0.01)	77(1.14)	0.3234
Sikh	421(1.32)	13(3.09)	03(0.71)	06(1.43)	02(0.48)	0(0)	02(0.48)	
Christian	482(1.51)	14(2.9)	05(1.04)	03(0.62)	02(0.41)	0(0)	04(0.83)	
Buddhist	316(0.99)	10(3.16)	02(0.63)	04(1.27)	01(0.32)	0(0)	03(0.95)	
Type								0.6358
R	31,338(98.14)	984(3.14)	172(0.55)	361(1.15)	130(0.42)	2(0.006)	319(1.02)	
V	593(1.86)	05(0.84)	01(0.17)	03(0.51)	01(0.17)	0(0)	0(0)	
Total	31,931	989	173	364	131	2	319	

*R* Replacement, *V* Voluntary.

## Electronic supplementary material

Below is the link to the electronic supplementary material.

Supplementary Material 1

## Data Availability

All data generated or analysed during this study are included in this article [and its supplementary file]. The raw data utilized in this study are available from the corresponding author upon reasonable request.
