# Supplementary material for: Prevalence and trends of transfusion transmitted infections among blood donors in a tertiary care hospital of Assam
Source: Sci Rep. 2025 Aug 16;15:30026. doi: 10.1038/s41598-025-97381-w (PMC12357952; doi:10.1038/s41598-025-97381-w)
Supplement: Supplementary file 1 — Supplementary Material 1 [file 41598_2025_97381_MOESM1_ESM.doc]

**Prevalence and Trends of Transfusion Transmitted Infections among Blood Donors in a Tertiary Care Hospital of Assam**

**Authors: ***Dr Madhumita Das^1^ MD, PhD, Dr Samim S. Hoque^2^ MD, Dr Argha Baruah^1^ MD

**Affiliation:** ^1^Guwahati Neurological Research Centre Institute of Medical Science,

North Guwahati, Assam, 781031

^2^Blood Centre, All India Institute of Medical Science, Guwahati, 781101.

**Table S1**: Blood group wise frequency distribution of donors with respect to TTIs positivity

| **TTI Status** | **A** | **AB** | **B** | **O** | **RhP** | **RhN** | ***p* value** |
| --- | --- | --- | --- | --- | --- | --- | --- |
| HBV Positive  (n=173)  Negative  (n=31758)  HCV Positive  (n=364)  Negative  (n=31567)  HIV Positive  (n=131)  Negative  (n=31800)  Syphilis Positive  (n=319)  Negative  (n=31612) | 38  (21.97)  7527  (23.7)  84  (23.08)  7481  (23.7)  33  (25.19)  7532  (23.69)  78  (24.45)  7487  (23.68) | 10  (5.78)  2342  (7.37)  27  (7.42)  2325  (7.37)  15  (11.45)  2337  (7.35)  32  (10.03)  2320  (7.34) | 49  (28.32)  9821  (30.92)  103  (28.3)  9767  (30.94)  42  (32.06)  9828  (30.91)  104  (32.6)  9766  (30.89) | 76  (43.93)  12068  (38.0)  150  (41.21)  11994  (37.99)  41  (31.3)  12103  (30.06)  105  (32.92)  12039  (38.08) | 169  (97.69)  30854  (97.15)  356  (97.8)  30667  (97.15)  129  (98.47)  30894  (97.15)  302  (94.67)  30721  (97.18) | 04  (2.31)  904  (2.85)  08  (2.2)  900  (2.85)  02  (1.53)  906  (2.85)  17  (5.33)  891  (2.82) | (O/AB)  <0.0001  <0.0001  0.002  0.0004 |
| Total Donor (n=31931) | 7565  (23.69) | 2352  (7.37) | 9870  (30.91) | 12144  (38.03) | 31023  (97.16) | 908  (2.84) |  |

**Table S2.** Prevalence of mixed TTI infections at GIMS Blood Centre from Jun’15 to Dec’23.

| TTI Status with Blood Group | Replacement | | Voluntary | | Total Donor  (31931) |
| --- | --- | --- | --- | --- | --- |
|  | Female | Male | Female | Male |  |
| HBV+HCV  A + ve  O - ve  O + ve  HBV+ Syphilis  A + ve  O + ve  HCV+HIV  A + ve  B +ve  O + ve  HCV+ Syphilis  AB +ve  B -Ve  HIV+ Syphilis  A + ve  AB +ve  B +ve  O + ve  HCV+HIV+ Syphilis  B +ve  Total | 0(0%)  0(0%)  0(0%)  0(0%)  0(0%)  0(0%)  0(0%)  0(0%)  0(0%)  0(0%)  0(0%)  0(0%)  0(0%)  0(0%)  0(0%)  0(0%)  0(0%)  0(0%)  0(0%)  0(0%)  0(0%)  0(0%) | 12(0.038%)  01(0.003%)  01(0.003%)  10(0.031%)  02(0.006%)  01(0.003%)  01(0.003%)  06(0.019%)  01(0.003%)  03(0.009%)  02(0.006%)  02(0.006%)  01(0.003%)  01(0.003%)  16(0.05%)  05(0.015%)  02(0.006%)  03(0.009%)  06(0.019%)  01(0.003%)  01(0.003%)  39(0.122%) | 0(0%)  0(0%)  0(0%)  0(0%)  0(0%)  0(0%)  0(0%)  0(0%)  0(0%)  0(0%)  0(0%)  0(0%)  0(0%)  0(0%)  0(0%)  0(0%)  0(0%)  0(0%)  0(0%)  0(0%)  0(0%)  0(0%) | 0(0%)  0(0%)  0(0%)  0(0%)  0(0%)  0(0%)  0(0%)  0(0%)  0(0%)  0(0%)  0(0%)  0(0%)  0(0%)  0(0%)  0(0%)  0(0%)  0(0%)  0(0%)  0(0%)  0(0%)  0(0%)  0(0%) | **12(0.038%)**  01(0.003%)  01(0.003%)  10(0.031%)  **02(0.006%)**  01(0.003%)  01(0.003%)  **06(0.019%)**  01(0.003%)  03(0.009%)  02(0.006%)  **02(0.006%)**  01(0.003%)  01(0.003%)  **16(0.05%)**  05(0.015%)  02(0.006%)  03(0.009%)  06(0.019%)  **01(0.003%)**  01(0.003%)  **39(0.122%)** |

**Table S3.** Multivariate analysis of the associations between transfusion-transmissible infections and demographic characteristics of the blood donors

| Characteristics | Positive for any TTIs | | | |
| --- | --- | --- | --- | --- |
|  | *t* Stat | *p* value | Lower 95% | Upper 95% |
| Gender  Age  Locality  Religion  Blood Group  Donor Type | -1.46  5.88  -2.49  1.09  -0.92  -1.49 | 0.14  <0.0001  0.01  0.28  0.36  0.14 | -5.48  0.02  -0.48  -0.08  -0.06  -2.47 | 0.81  0.05  -0.06  0.29  0.02  0.34 |
